# Substance P-Mediated Vascular Protection Ameliorates Bone Loss

**DOI:** 10.1155/2023/9903336

**Published:** 2023-04-29

**Authors:** Doyoung Kim, Jiyuan Piao, Jeong Seop Park, Dahyun Lee, Dae Yeon Hwang, Hyun Sook Hong

**Affiliations:** ^1^Department of Biomedical Science and Technology, Graduate School, Kyung Hee University, 1 Hoegidong, Dongdaemun-gu, Seoul 02447, Republic of Korea; ^2^Kyung Hee Institute of Regenerative Medicine (KIRM), Medical Science Research Institute, Kyung Hee University Medical Center, Republic of Korea; ^3^East-West Medical Research Institute, Kyung Hee University, 1 Hoegidong, Dongdaemun-gu, Seoul 02447, Republic of Korea

## Abstract

Estrogen deficiency causes bone loss via diverse pathological cellular events. The involvement of the vasculature in bone formation has been widely studied, and type H vasculature has been found to be closely related to bone healing. Ovariectomy- (OVX-) induced estrogen deficiency reduces type H vessel density and promotes reduction of bone density. Analysis of early events after OVX showed that estrogen deficiency preferentially induces oxidative stress, which might provoke endothelial dysfunction and reduce angiogenic factors systemically and locally. The instability of the vascular potential is expected to promote bone loss under estrogen deficiency. Substance P (SP) is an endogenous neuropeptide that controls inflammation and prevents cell death under pathological conditions. SP can elevate nitric oxide production in endothelial cells and inhibit endothelial dysfunction. This study is aimed at investigating the preventive effects of systemically injected SP on OVX-induced vascular loss and osteoporosis onset. SP was systemically administered to OVX rats twice a week for 4 weeks, immediately after OVX induction. OVX conditions could decrease antioxidant enzyme activity, type H vessels, and angiogenic growth factors in the bone marrow, followed by inflammation and bone loss. However, pretreatment with SP could block type H vessel loss, accompanied by the enrichment of nitric oxide and sustained angiogenic factors. SP-mediated early vascular protection inhibits bone density reduction. Altogether, this study suggests that early administration of SP can block osteoporosis development by modulating oxidative stress and protecting the bone vasculature and angiogenic paracrine potential at the initial stage of estrogen deficiency.

## 1. Introduction

Osteoporosis is the most common type of age-related metabolic bone disease and a multifactorial disorder that eventually contributes to bone loss. One in three women and one in five men over the age of 50 will suffer a broken bone due to osteoporosis. In women, menopause is a primary cause of osteoporosis. Up to 20% of bone loss occurs during menopause. After this interval of relatively rapid bone loss, bone density decreases about 0.5% per year. By age 80 years, women have lost, on average, approximately 30% of their peak bone mass [1, 2]. Since estrogen prevents bone weakening by slowing the natural breakdown of bone, its reduction during menopause is believed to contribute to bone loss [3].

There are many treatments available to prevent bone loss, including bisphosphonates, denosumab, and hormone-related therapy. These treatments are primarily aimed at inhibiting osteoclast activation or promoting osteoblast proliferation. However, they are not ideal treatments to block disease development and have serious side effects such as back pain and elevation of blood pressure. Thus, the administration of medications for osteoporosis is constantly controlled, depending on the patient's condition.

Blood vessels serve as a supportive source of mesenchymal stem cells that differentiate into osteoblasts [4–6]. During bone development and regeneration, the migration of osteogenic precursor cells to the bone defect area is closely related to the invasion of blood vessels [7]. Age-related decline in vascular endothelial function has been clearly observed under estrogen-deficient conditions [8, 9]. Thus, a key role for blood vessels in bone regeneration is widely expected in the aged population, and angiogenic–osteogenic coupling is considered crucial for bone homeostasis.

Angiogenic–osteogenic coupling is tightly regulated by a specific capillary in the bone, known as a type H vessel with high expression of CD31 and endomucin [6, 10]. These type H vessels are known to mediate the growth of the bone vasculature, maintain perivascular osteoprogenitors, and enhance osteogenesis [10, 11]. The osteogenic progenitor cells surrounding type H vessels express the transcription factors osterix and Runx2, which promote bone formation [7]. Type H vascular endothelium secretes growth factors that are closely related to the proliferation and survival of bone progenitor cells [12]. Thus, type H vessels are anticipated to act as important promoters of bone regeneration at bone defect sites [7]. The density of type H vessels is most abundant in the young/growing organism and decreases with age. Indeed, osteoporotic conditions show deficiency of type H vessels in the bone marrow (BM) from an early stage of osteoporosis. This suggests that type H vessels can be used as sensitive biomarkers of bone mass [6].

Vascular endothelial growth factor (VEGF), platelet-derived growth factor-BB (PDGF-BB), and slit guidance ligand 3 have been widely investigated for their ability to promote vascularization during bone formation. They are mostly produced in vascular cells, exerting their role in an autocrine or paracrine way. These growth factors can stimulate endothelial cells, endothelial progenitor cells (EPC), and mesenchymal stem cell migration to couple angiogenesis and osteogenesis, leading to an increase in the number of type H vessels and enhanced bone formation [11, 13, 14]. In the osteoporotic animal model with ovariectomy (OVX), concentrations of serum and BM PDGF-BB, VEGF, and CD31^hi^Endomucin^hi^ type H vessels were significantly decreased [15–17]. Maintenance of PDGF-BB at high concentration locally or systemically could enhance CD31^hi^Endomucin^hi^ type H vessels, leading to an increase in bone volume [11]. Disruption of estrogen-regulated PDGF-BB signaling in OVX has been reported to result in microvessel destabilization, capillary rarefaction, increased vascular permeability, and aberrant angioarchitecture [18]. That is, estrogen deficiency can pervert angiogenic signaling to cause endothelial dysfunction, accompanied by the lack of angiogenic soluble factors. Therefore, preservation of soluble factor-mediated angiogenic signaling is surmised to contribute to osteogenesis by providing vascular stability and stem cell engagement under estrogen deficiency. The maintenance of angiogenic factors is anticipated to require the protection of its cellular source under estrogen deficiency-induced stress.

The exact mechanism for the loss of vascular potential due to estrogen deficiency is not clear; however, estrogen exerts antioxidant effects on the endothelium by modulating NADPH oxidase (NOX) expression and superoxide production [19]. Moreover, estrogen induces the generation of nitric oxide (NO) via activation of Akt and endothelial nitric oxide synthase (eNOS) [20]. NO is responsible for positive regulation of vascular tone, angiogenesis, mitogenesis, and inflammation in the endothelium. Thus, the lack of estrogen may cause an imbalance between free radicals/reactive oxygen species (ROS) and antioxidants through excessive ROS accumulation and the low bioavailability of NO, which causes cell death and inflammation. Lean et al. corroborated the idea that OVX conditions alter the generation of ROS and the antioxidant defense capacity of the cell, leading to an accumulation of ROS, which stimulates the production of tumor necrosis factor- (TNF-) *α* in the BM at the early phase after OVX induction [21, 22]. Taken together, estrogen deficiency is thought to provoke oxidative stress, leading to cellular damage and inflammation, followed by bone loss.

To inhibit oxidative stress-induced cellular damage, the production of ROS should be restricted from the early stage of estrogen deficiency. However, the sources of ROS vary, and their production is mediated by complicated cellular responses. Thus, complete control of ROS production is difficult in vivo. Few studies have investigated the direct action of antioxidants on the activity of bone cells [23]. Moreover, expression of the estrogen receptor is known to be decreased in the aged/postmenopausal status, indicating weak efficacy of exogenous estrogen supplementation [24, 25]. Thus, activation of cellular survival-related signaling or NO enrichment at the early phase after estrogen loss is anticipated to be strategy to protect vascular cells and tissues against oxidative stress.

Despite the importance of oxidative stress and vasculature injury during bone loss, investigations into the temporal analysis of the occurrence of oxidative stress, vascular dysfunction, and bone loss are currently scarce. If the estrogen deficiency-mediated pathological events are analyzed over time, the therapeutic target can be figured out and used to block the development of osteoporosis.

Substance P (SP) is an endogenous neuropeptide capable of stimulating stem cell mobilization, proliferation, and protecting the endothelium against oxidative stress and inflammation, accompanied by NO production [26–31]. In previous studies, SP was administered to OVX animals with excessive inflammation and reduced bone density to evaluate the inhibitory effect of SP on additional bone loss. SP can alleviate bone density reduction by modulating the development of regulatory T cells and suppressing inflammation in OVX animal [17]. Moreover, SP treatment elevated the production of VEGF and PDGF-BB in stem cells in vitro and in a diabetic or nondiabetic wound model in vivo [26, 27, 32, 33]. Thus, the early application of SP after OVX-induced estrogen loss was inferred to prevent the onset of bone loss, possibly by enriching NO/angiogenic factors and preserving the angioarchitecture in OVX rats.

This study explored the progression of occurrence for oxidative stress, vascular injury, inflammation, and bone loss over time using an OVX animal model and evaluated the preventive effect of SP, a neuropeptide, on loss of vascular potential and bone mass in OVX animals by analyzing serum/BM aspirate biochemical markers, tissue histology, and bone density in vivo and evaluated the protective effect of SP on BM-derived EPCs against oxidative stress in vitro.

## 2. Materials and Methods

### 2.1. Induction of Osteoporosis

Six-week-old female Sprague Dawley (SD) rats (160–170 g) were purchased from DBL (Daehan Bio Link, Seoul, Korea). All animals were housed in an animal-holding room under a regular light/dark cycle. All the animals were fed a standard chow diet. This study was approved by the Ethical Committee for Experimental Animals of Kyung Hee University Hospital (approval number: KHMC-IACUC-E18-009, KHMC-IACUC-22-011).

After a 1-week adaptation period, osteoporosis was induced as previously described [17]. Briefly, female SD rats were anesthetized using intraperitoneal injections of ketamine (100 mg/kg, Yuhan, Seoul, Korea) and Rompun (1.2 mg/kg, Bayer Healthcare, Kyunggi-do, Korea). Hair at the surgical site was removed and a 2 cm skin incision was made on both sides of the abdomen. After the periovarian adipose tissue and ovaries were pulled out, the ovaries were ligated with 5-0 silk and removed. Both sides of the muscle and skin were sutured with a 3-0 silk. Rats were randomly distributed into three groups: (1) sham, (2) OVX+saline, and (3) OVX+SP.

### 2.2. Administration of SP

The SP (Sigma-Aldrich, St. Louis, MO, USA) was diluted in saline (JW Pharmaceutical, Seoul, Korea) immediately before use and administrated intravenously twice a week for 4 weeks at a dose of 5 nmol/kg. Saline was used as a control vehicle.

### 2.3. Measurement of Bone Density Using Ex Vivo Micro-Computed Tomography (*μ*-CT)

Femurs harvested from SD rats were scanned using a *μ*-CT scanner (Skyscan 1173 X-ray microtomography; Bruker, Billerica, MA, USA) at a resolution of 13.85 *μ*m to evaluate the degree of osteoporosis, as previously described [17]. In brief, after three-dimensional reconstruction, the bone mineral density, bone volume fraction, and trabecular bone space in the metaphysis area were calculated using CT analysis software.

### 2.4. Histological Analysis

Femurs were isolated and fixed in 3.7% formaldehyde (Sigma-Aldrich). Samples were decalcified with decalcifying solution (Sigma-Aldrich) and processed with a TP1020 tissue processor (Leica Biosystems, Wetzlar, Germany) to prepare paraffin blocks, and 5.0 *μ*m thick sections were prepared. For trichrome staining, a NovaUltraTM Masson trichrome stain kit (IHC World, Woodstock, MD, USA) was used. For immunohistochemistry staining, the VECTASTAIN ABC kit or ABC-AP kit (Vector Laboratories, Burlingame, CA, USA) was used. Briefly, rehydrated samples were boiled with 0.01 M sodium citrate (Sigma-Aldrich) for antigen retrieval. For blocking activity of endogenous hydrogen peroxidase, the samples were treated with 0.5% H_2_O_2_. Next, the samples were permeabilized with 0.3% Triton X-100 (Sigma-Aldrich). For blocking nonspecific binding of antibodies, the samples were incubated with 2% normal horse serum for 1 h at room temperature (RT) and then treated with primary antibodies against CD31, endomucin. The samples were incubated with biotinylated secondary antibodies for 1 h at RT, followed by incubation with an avidin–biotin complex solution. The substrate solution, ImmPACT NovaRed or Vector Blue (Vector Laboratories), was used to visualize the reactive area in the tissue. Finally, samples were counterstained with Fast Red (Vector Laboratories).

### 2.5. Preparation of Protein Extracts and Western Blot Analysis

The femur biopsies were flushed with phosphate-buffered saline (PBS; WELGENE, Daegu, Korea) three times and centrifuged at 1,500 rpm (362 g) for 5 m. The supernatant was isolated for cytokine analysis and the cell pellet (bone marrow aspirates, BMA) was treated with lysis buffer (Cell Signaling Technology, Danvers, MA, USA) and 2 mM PMSF (Roche, Basel, Switzerland). The lysed sample was centrifuged at 12,000 rpm (13572 g) for 10 m, and the supernatants were collected. The protein concentrations were determined using a bicinchoninic acid protein assay kit (Thermo Fisher Scientific, Rockford, IL, USA). Protein lysates were separated using sodium dodecyl sulfate–polyacrylamide gel electrophoresis and transferred to nitrocellulose (GE Healthcare, Amersham, UK) or polyvinylidene difluoride (Pall Corporation, New York, USA) membranes. The membrane was blocked with 5% skim milk (Becton, Dickinson and Company, Franklin Lakes, NJ, USA) containing bovine serum albumin (Sigma-Aldrich) and then incubated with primary antibodies for CD31, glyceraldehyde 3-phosphate dehydrogenase (GAPDH; Abcam, Cambridge, UK), eNOS, and p-eNOS (Cell Signaling Technology), followed by anti-immunoglobulin G horseradish peroxidase-conjugated secondary antibodies for 1 h at room temperature. Membranes were developed using EZ-Western Lumi Pico (DoGenBio, Seoul, Korea) or WESTAR ETA C ULTRA 2.0 (Cyanagen, Bologna, Italy).

The expression levels were analyzed and quantified using ImageJ software (version 1.52a).

### 2.6. Enzyme-Linked Immunosorbent Assay (ELISA)

The concentrations of VEGF, PDGF-BB, interleukin- (IL-) 10 (R&D Systems, Minneapolis, MN, USA), and TNF-*α* (BioLegend Inc., San Diego, CA, USA) in serum and BM were measured using ELISA according to the manufacturer's instructions. Optical density was measured at 450 nm using an EMax Endpoint ELISA Microplate Reader (Molecular Devices, Sunnyvale, CA, USA).

### 2.7. Measurement of Superoxide Dismutase (SOD) Activity

Bone marrow aspirates (BMA) were prepared by flushing the BM with PBS, and the total protein was isolated. SOD activity in the BMA of rats was determined using a SOD activity assay kit (Abcam). The protein lysates of BMA were mixed with WST and enzyme working solutions. After incubation at 37°C for 20 m, the absorbance was measured at 450 nm using the EMax Endpoint microplate reader (Molecular Devices).

### 2.8. Measurement of NO Concentration

The amount of NO in the BMA was measured using the Griess reagent system (Promega, Madison, WI, USA). The samples were added to the wells and incubated with sulfanilamide solution for 10 m at RT. Next, the N-1-napthylethylenediamine dihydrochloride solution was added to the wells and incubated for 10 m at RT. The optical density was measured at 540 nm using the EMax Endpoint microplate reader (Molecular Devices). The concentration of NO was then quantified.

### 2.9. BM-EPC Culture

Human BM mononuclear cells (BM-MNCs) were obtained from Stem Cell Technology (Vancouver, Canada). BM-derived MNCs were seeded in culture dishes coated with human fibronectin (Sigma-Aldrich) and cultured in endothelial growth media-2 (Lonza, Basel, Switzerland). The medium was changed once every alternate day. BM-derived EPCs were subcultured at 80% confluence. To examine the protective effect of SP on EPC under oxidative stress, SP (final concentration: 100 nM) was added to EPC twice at 30 min intervals and then treated with 300 *μ*M hydrogen peroxide (H_2_O_2_) for 8 h. The effects of pretreated SP on EPC under oxidative stress were determined.

### 2.10. Statistical Analysis

All data are presented as mean ± standard deviation. *p* < 0.05 was considered statistically significant. Statistical analysis of the data was performed using unpaired, two-tailed Student's *t*-tests.

## 3. Results

### 3.1. Estrogen Deficiency Causes Vascular Loss, Followed by Bone Loss

**Figure 1:**
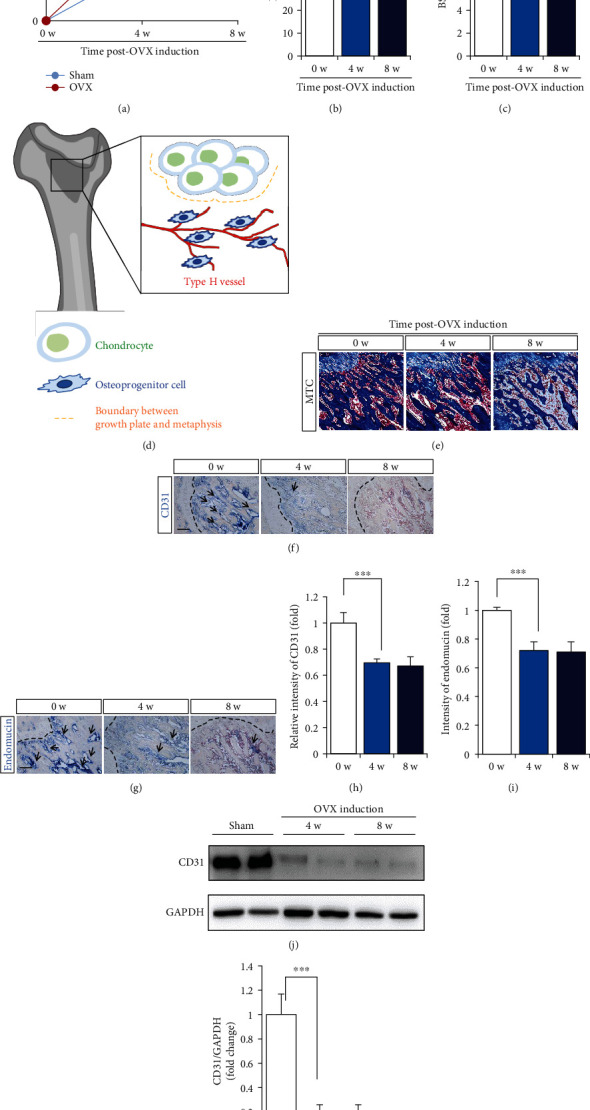
Ovariectomy (OVX) causes bond loss and the lack of type H vessel. (a) Change of rat body weight at 0, 4, and 8 weeks post-OVX induction. (b, c) Micro-CT analysis for quantification of bone density was determined at 0, 4, and 8 weeks postinduction. (d) Schematic diagram of microenvironment of bone metaphysis region. (e) The representative images of Masson's Trichrome (MTC). Expression of CD31 (f) and endomucin (g) in femoral metaphysis regions was determined by IHC staining. The dotted lines indicate boundary between growth plate and metaphysis. Scale bar: 100 *μ*m. (h, i) Quantification for intensity of CD31 and endomucin staining was performed. (j, k) Western blot analysis of CD31 expression in BMA at 0, 4, and 8 weeks postinduction was performed. GAPDH was used as internal loading control and normalized for quantitative analysis. Values are mean ± standard deviation (SD), ^∗∗^*p* < 0.01, and ^∗∗∗^*p* < 0.001. BV: trabecular bone volume; TV: tissue volume; BS: bone surface; n.s.: not significant.

To check the occurrence of vascular injury and bone loss after OVX induction by time, OVX was induced and the change in bone density and type H vessel in the BM was monitored for 8 weeks postinduction. OVX-induced estrogen deficiency facilitated an increase in body weight, and a distinct difference was observed at 4 weeks post-OVX induction (Figure 1(a)). Analysis of bone density by micro-CT revealed that bone density was reduced at 8 weeks, and there was no significant change at 4 weeks (Figures 1(b) and 1(c)). MTC staining showed that as the bone structure decreased, fat tissue increased, which was apparent at 8 weeks (Figures 1(d) and 1(e)). This was consistent with the micro-CT data.

To examine vascular injury in OVX rats, type H vessels were examined. The type H vessels were identified at specific locations, mainly in the metaphysis near the growth plate (Figure 1(d)).

The endothelium of type H vessels is strongly positive for the endothelial cell-surface markers CD31 and endomucin. Histological analysis revealed that the loss of CD31^+^ or endomucin^+^ vessels near the growth plate (black dotted line) was clearly detected at 4 weeks, which corresponds to the condition of normal bone density (Figures 1(f) and 1(g), Supplementary Figure 1). Staining intensity for CD31 or endomucin was quantified (Figures 1(h) and 1(i)). Endomucin is expressed in vascular endothelial and hematopoietic stem cells. Therefore, examination of the protein level of endomucin with whole BM aspirates was not suitable to infer type H vessel density. Accordingly, western blot analysis for expression of CD31 in BM aspirates was carried out, showing an immoderate reduction from 4 weeks post-OVX induction (Figures 1(j) and 1(k)).

This result revealed that OVX causes a deficiency of CD31^+^ and endomucin^+^ type H vessels at 4 weeks post-OVX induction and then provokes bone density reduction from 8 weeks postinduction.

### 3.2. Estrogen Deficiency Induces the Reduction of Angiogenic Growth Factors in Serum and BM and Then Creates Systemic Inflammation by Elevating TNF-*α* and Decreasing IL-10

**Figure 2:**
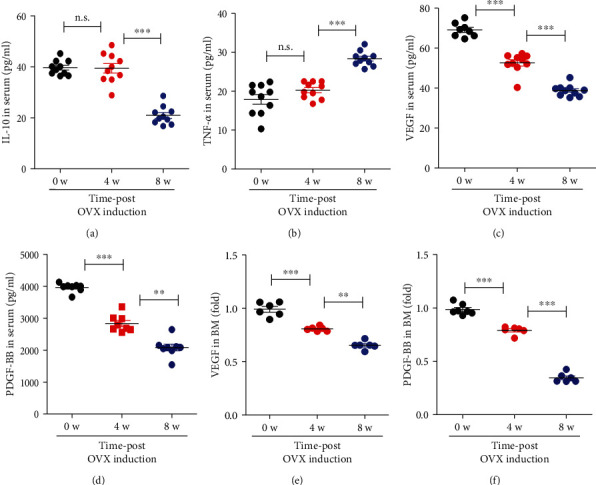
OVX creates the lack of angiogenic potential and proinflammatory condition. The amounts of IL-10 (a), TNF-*α* (b), VEGF (c), and PDGF-BB (d) in serum at 0, 4, and 8 weeks postinduction were measured using ELISA. The amounts of VEGF (e), PDGF-BB (f) in BM at 0, 4, and 8 weeks post-OVX induction were measured using ELISA. Values are mean ± SD, ^∗∗^*p* < 0.01, and ^∗∗∗^*p* < 0.001. n.s.: not significant.

Vascular loss is expected to occur because of the instability of vascular component cells due to insufficient angiogenic growth factors or excessive inflammation. As shown in Figure 1, a reduction in type H vessels was observed at 4 weeks post-OVX induction. Next, inflammatory and angiogenic factors were analyzed systemically and locally. To check the inflammatory condition, IL-10, an anti-inflammatory cytokine, and TNF-alpha, a pro-inflammatory cytokine in serum, were quantified using ELISA. Alterations in the levels of IL-10 and TNF-*α* in serum were seldom detected at 4 weeks, but change was clearly observed at 8 weeks, with a decrease in IL-10 and an increase in TNF-*α* (Figures 2(a) and 2(b)). IL-17, a crucial inflammatory factor in bone loss, was also elevated at 8 weeks, but not at 4 weeks after OVX induction (Supplementary Figure 2). These data indicate the systemic occurrence of inflammatory conditions, which might be related to the initiation of bone loss at 8 weeks post-OVX induction. By contrast, the concentrations of VEGF and PDGF-BB, the representative angiogenic growth factors, clearly decreased after 4 weeks, and this difference increased at 8 weeks (Figures 2(c) and 2(d)). Considering that 8 weeks post-OVX induction was characterized by marked bone loss and inflammation, the systemic loss of angiogenic factors is estimated to occur prior to inflammation and reduction in bone density.

To determine the level of angiogenic factors in the BM environment, the amount of VEGF and PDGF-BB in the BM was examined using ELISA (Figures 2(e) and 2(f)). The reduction in VEGF and PDGF-BB levels was detected at 4 weeks post-OVX induction, consistent with serum levels.

These data imply that OVX induction creates an angiogenic factor–deficient environment locally and systemically within 4 weeks and separately causes inflammatory conditions within 8 weeks of OVX induction. Insufficient angiogenic factors in the serum and BM are thought to be involved in the loss of type H vessels in the BM. This early stage is expected to facilitate the development of osteoporosis.

### 3.3. Treatment with SP Blocks the Loss of Angiogenic Potential by Preserving Vascular Factors and Type H Vessels Early after OVX Induction

**Figure 3:**
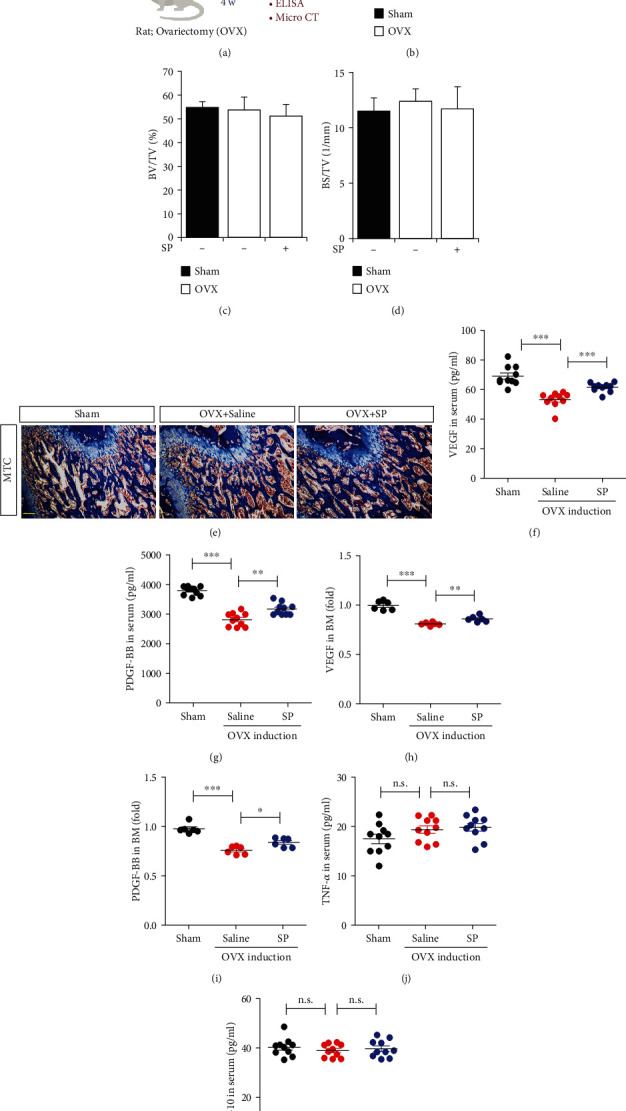
Systemic administration of SP prevents loss of angiogenic growth factors due to OVX within 4 weeks. (a) Experiment scheme for OVX and SP treatment. SP was injected intravenously twice a week for 4 weeks. Saline was used as vehicle control. (b) Change of rat body weight at 4 weeks was assessed. (c, d) Micro-CT analysis for quantification of bone density at 4 weeks post-OVX induction was carried out. (e) The representative images of Masson's Trichrome staining of the femur. Scale bar: 100 *μ*m. The amounts of VEGF (f), PDGF-BB (g), TNF- *α* (j), and IL-10 (k) in serum were measured by ELISA. The amounts of VEGF (h) and PDGF-BB (i) in BM were measured by ELISA. Values are mean ± standard SD, ^∗^*p* < 0.05, ^∗∗^*p* < 0.01, and ^∗∗∗^*p* < 0.001. BV: trabecular bone volume; TV: tissue volume; BS: bone surface; n.s.: not significant.

**Figure 4:**
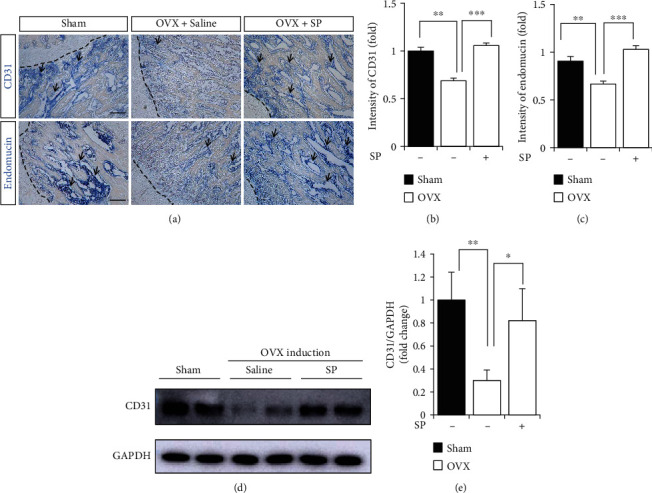
SP treatment blocks the deficiency of type H vessel from OVX-induced impairment. (a) The representative images of IHC staining for CD31 and endomucin in femoral metaphysis regions. The dotted lines indicate boundary between growth plate and metaphysis. Scale bar: 100 *μ*m. (b, c) Quantification for intensity of CD31 and endomucin staining was performed. (d, e) Western blot analysis for CD31 expression using BMA at 4 week was performed. GAPDH was used as internal loading control and normalized for quantitative analysis. Values are mean ± SD, ^∗^*p* < 0.05, ^∗∗^*p* < 0.01.

To investigate the protective effect of SP on angiogenic potential and bone mass, SP was intravenously administered to OVX rats for 4 weeks, and then, serum and BM were analyzed for angiogenic/inflammatory factors, type H vessels, and bone mass (Figure 3(a)). Early treatment with SP alleviated the gain in body weight at 4 weeks (Figure 3(b)). At this time, distinct bone loss did not occur, and the concomitant effect of SP treatment on bone density was rarely observed (Figures 3(c) and 3(d)). This was confirmed via the histological analysis (Figure 3(e)). Angiogenic factors, VEGF and PDGF-BB, were decreased by OVX induction, but SP treatment clearly inhibited their reduction in serum and BM (Figures 3(f)–3(i)). Four weeks post-OVX induction, systemic inflammation was not provoked, and SP treatment did not affect its condition (Figures 3(j) and 3(k)). To evaluate type H density in BM, CD31 or endomucin expression was assessed (Figure 4). As predicted, OVX induction decreased the density of CD31^+^ or endomucin^+^ type H vessels (Figures 4(a)–4(c)). SP treatment blocked the disappearance of type H vessels in the BM at 4 weeks post-OVX induction. SP-mediated endothelial protection was confirmed using western blot of CD31 expression (Figures 4(d) and 4(e)).

Collectively, the administration of SP for 4 weeks mitigated OVX-mediated vascular loss by enriching angiogenic growth factors and type H vasculature.

### 3.4. SP Rescues Type H Vessel and Then Blocks Bone Loss

Systemic administration of SP can sustain angiogenic potential by blocking the loss of growth factors and type H vessels under estrogen deficiency. Next, we examined whether SP-enhanced angiogenic potential is related to the inhibition of bone mass.

**Figure 5:**
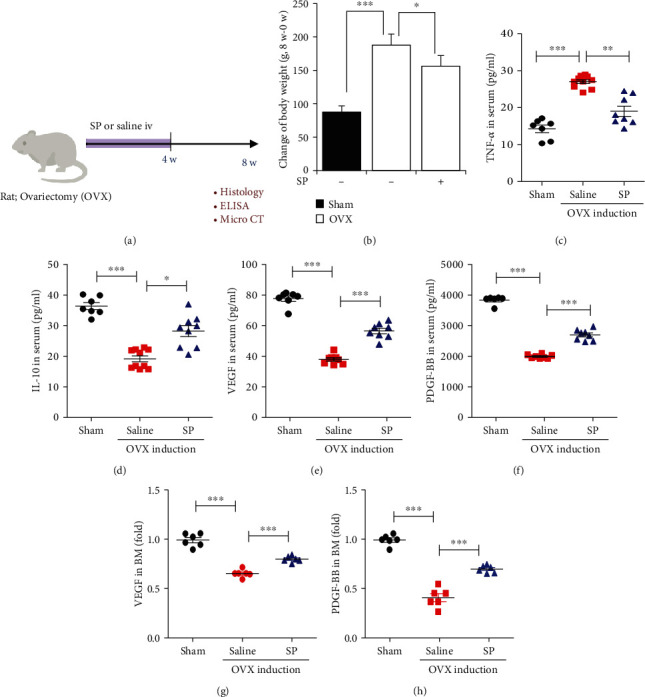
The early treatment of SP inhibits the loss of angiogenic growth factor and ameliorates systemic inflammation at 8 weeks post-OVX induction. (a) Experiment scheme for OVX and SP treatment. SP was injected intravenously twice a week for 4 weeks, and it was stopped until 8 weeks post-OVX induction. Saline was used as vehicle control. (b) Change of rat body weight at 8 weeks post-OVX induction. The amounts of TNF*α* (c), IL-10 (d), VEGF (e), and PDGF-BB (f) in serum were measured by ELISA. The amounts of VEGF (g) and PDGF-BB (h) in BM were measured by ELISA. Values are mean ± SD, ^∗^*p* < 0.05, ^∗∗^*p* < 0.01, and ^∗∗∗^*p* < 0.001.

SP was injected into OVX animals for 4 weeks, and then, the OVX animals were maintained without extra treatment until 8 weeks. Eight weeks post-OVX induction, growth factors, vasculature, and bone mass were examined (Figure 5(a)).

OVX increased the body weight at 8 weeks, which was higher than that at 4 weeks. However, early treatment with SP alleviated this change at 8 weeks post-OVX induction (Figure 5(b)). Consistent with previous data, 8 weeks after OVX induction caused systemic inflammation by elevating TNF-*α* and decreasing IL-10 (Figures 5(c) and 5(d)). OVX-induced inflammation was reversed via SP treatment for 4 weeks immediately after OVX induction. Angiogenic factors, VEGF and PDGF-BB, declined excessively at 8 weeks post-OVX induction (Figures 5(e)–5(h)).

However, SP treatment sustained considerable levels of VEGF and PDGF-BB in the serum and BM. This effect of SP on the level of angiogenic/inflammatory soluble factors at 8 weeks was attributed to treatment with SP for 4 weeks after OVX induction. Eight weeks post-OVX induction is 4 weeks to the day following the last injection of SP. Thus, this effect of SP might be the result of a cascade response of cell/tissue that was affected by SP over the 4 weeks after OVX induction.

**Figure 6:**
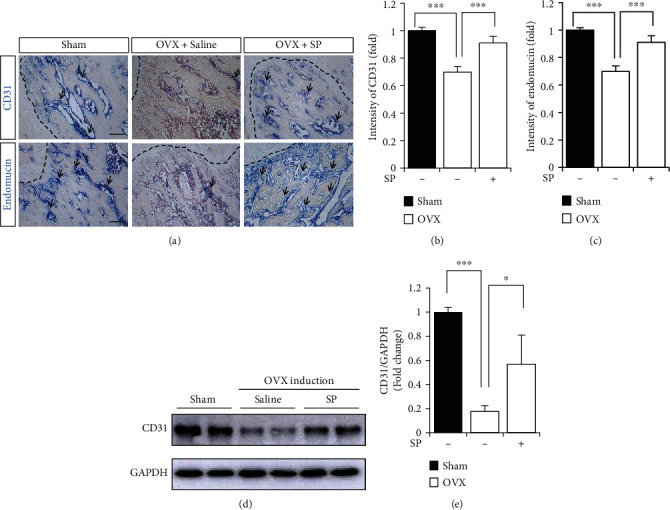
Early protection of type H vessel by SP contributes to the preservation of type H vessel at 8 weeks post-OVX. (a) The representative images of IHC staining for CD31 and endomucin in femoral metaphysis regions. The dotted lines indicate boundary between growth plate and metaphysis. Scale bar: 100 *μ*m. (b, c) Quantification for intensity of CD31 and endomucin staining was performed. (d, e) Western blot analysis of CD31 expression of BMA at 8 weeks post-OVX induction. GAPDH was used as internal loading control and normalized for quantitative analysis. Values are mean ± SD, ^∗^*p* < 0.05, ^∗∗^*p* < 0.01.

**Figure 7:**
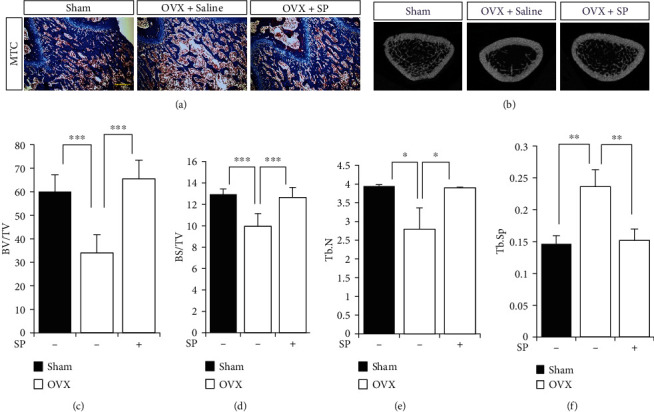
SP-mediated vascular protection mitigates bone density reduction. (a) The representative images of Masson's Trichrome (MTC) staining of femoral metaphysis regions. Scale bar: 100 *μ*m. (b) Representative images of femoral *μ*CT (c–f). Quantitative analysis of the trabecular bone fraction of the femur at 8 weeks post-OVX induction. Values are mean ± SD, ^∗^*p* < 0.05, ^∗∗^*p* < 0.01, and ^∗∗∗^*p* < 0.001. BV: trabecular bone volume; TV: tissue volume; BS: bone surface; Tb.N: trabecular number; Tb.SP: trabecular separation.

The distribution of type H vessels in the BM was analyzed (Figure 6). CD31^+^ or endomucin^+^ type H vessels rapidly disappeared in the OVX condition from 4 weeks, and its trend was aggravated at 8 weeks. However, SP injection protected the type H vessels against OVX-induced stress (Figures 6(a)–6(c)). The analysis of CD31 expression in the BMA also confirmed the effect of SP on the endothelium in the BM (Figures 6(d) and 6(e)). More importantly, SP-mediated early vascular protection inhibited bone loss progression (Figure 7). Histological analysis corroborated SP-mediated inhibition of bone loss (Figure 7(a)). Micro-CT analysis revealed that SP-treated OVX rats showed higher bone volume and number than saline-treated OVX rats (Figures 7(b)–7(f)). This result corroborates the finding that systemic administration of SP can preserve angiogenic potential that is impaired by OVX, and early protection of vasculature is able to block the progression of bone loss to maintain bone density similar to the non-OVX control.

### 3.5. SP Treatment Modulates Oxidative Stress by Enhancing SOD Activity and NO Production

This study showed that SP treatment inhibited the deficiency of angiogenic factors and type H vessels in OVX rats. However, it is unclear how SP protects vasculature. Oxidative stress is a condition in which the generation of ROS exceeds the capacity of the antioxidant defense system. Oxidative stress can occur as a consequence of excess generation of ROS, depressed antioxidant capacity, or a combination of these factors [34–37]. Uncontrolled ROS levels can reduce NO bioavailability by inducing eNOS uncoupling and promoting peroxynitrite generation, which causes cell apoptosis. By contrast, NO decreases ROS accumulation by inhibiting SOD inactivation. Thus, NO and ROS act as reciprocal inhibitors. Estrogen loss induces ROS accumulation and reduces NO production, leading to oxidative stress.

Loss of type H vessels was observed 4 weeks post-OVX induction. To examine the development of oxidative stress in the BM environment after OVX induction, SOD activity and NO concentration were determined using BMA at 3 and 4 weeks post-OVX induction.

**Figure 8:**
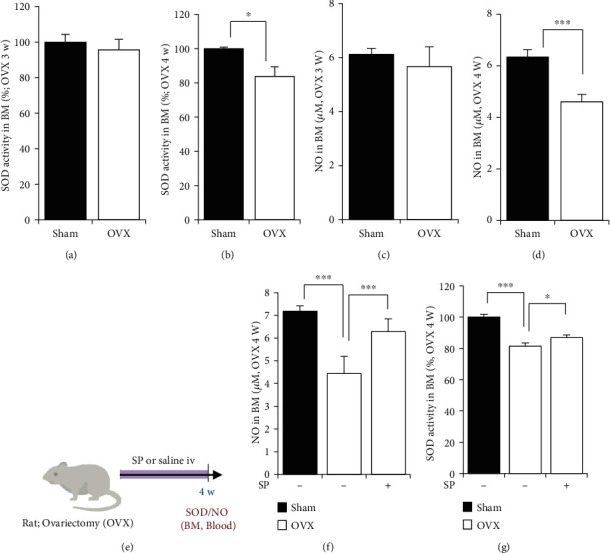
SP treatment reduces oxidative stress due to estrogen deficiency at 4 weeks post-OVX. SOD activity in BMA was measured at 3 (a) and 4 (b) weeks post-OVX induction. NO concentration in BMA was measured by the Griess reagent system at 3 (c) and 4 (d) weeks. (e) Experiment scheme for OVX and SP treatment. SP was injected intravenously twice a week for 4 weeks. Saline was used as vehicle control. NO concentration (f) and SOD activity (g) in BM at 4 week post-OVX induction were determined.

SOD activity and NO levels tended to decrease to an extent at 3 weeks post-OVX induction, but the difference was not statistically significant (Figures 8(a) and 8(c)). At 4 weeks post-OVX induction, which corresponds to a reduction in type H vessel density, SOD activity and NO levels were clearly reduced, indicating the occurrence of oxidative stress (Figures 8(b) and 8(d)). This change was observed more clearly 8 weeks after OVX induction (Supplementary Figure 3). SP treatment for 4 weeks after OVX induction resulted in elevated NO levels and enhanced SOD activity, compared to the nontreated OVX group (Figures 8(e)–8(g)). That is, SP treatment created NO-enriched conditions and enhanced antioxidant activity in the BM at 4 weeks post-OVX induction.

These data revealed the oxidative stress-induced cellular risk is perceived beyond 3 weeks, and cell damage actually happens at 4 weeks after OVX induction. Considering the critical role of NO in vasculature and inflammation, SP-induced type H protection might occur through sufficient generation of NO and restoration of SOD activity in the early phase of estrogen deficiency.

### 3.6. SP Supports Cell Survival against Oxidative Stress, Accompanied by Enrichment of NO In Vitro

This study confirms the early occurrence of oxidative stress in the BM of OVX rats. Oxidative stress can negatively affect the survival of diverse cells, including endothelial cells residing in the BM. SP treatment was expected to block oxidative stress and preserve type H vessels in the BM of OVX mice, as shown in Figures 4 and 8. The vasculature in bone appears to be formed mainly by vasculogenesis and angiogenesis [10, 38].

**Figure 9:**
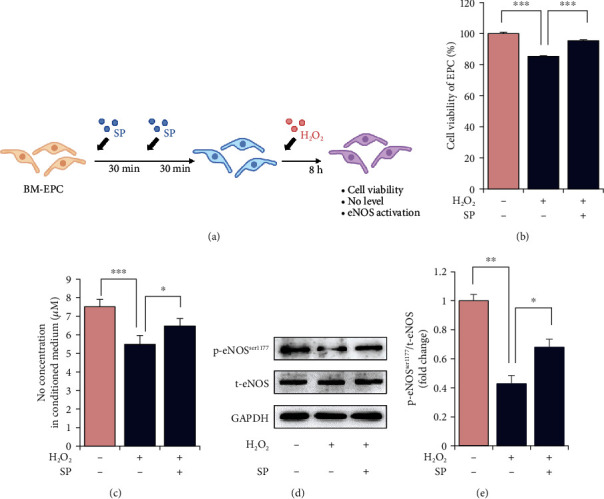
SP blocks oxidative stress-induced cellular impairment by enriching NO. (a) Experiment scheme for SP and H_2_O_2_ treatment on human bone marrow- (BM-) derived endothelial progenitor cells (EPCs). Saline was used as vehicle control. In order to examine the protective effect of SP on EPC against oxidative stress, SP was added to EPC twice at 30 m intervals, and then, H_2_O_2_ was treated for 8 h. (b) Cell viability of BM-EPCs was measured by WST assay. (c) NO concentration in BM-EPC conditioned medium was measured by the Griess reagent system. (d, e) Western blot analysis of p-eNOS and t-eNOS in BM-EPCs was performed. GAPDH was used as internal loading control. Values are mean ± SD, ^∗^*p* < 0.05, ^∗∗^*p* < 0.01, and ^∗∗∗^*p* < 0.001.

Next, we examined whether SP could protect BM EPCs that play a role in blood vessel formation under oxidative stress in vitro. SP was treated with EPC, and hydrogen peroxide was added to induce excessive ROS accumulation for 8 h (Figure 9(a)). The schedule of SP treatment was determined by referring to the in vivo administration of SP. As predicted, hydrogen peroxide treatment for 8 h reduced cell viability in a dose-dependent manner, and the final concentration was determined to be 300 *μ*M, with approximately 80% cell viability (Supplementary Figure 4). ROS-induced reduction in cell viability was blocked by pretreatment with SP (Figure 9(b)). At this time, eNOS phosphorylation and NO production were decreased by hydrogen peroxide treatment, but SP-pretreated cells maintained higher eNOS phosphorylation and NO concentrations than nontreated cells (Figures 9(c)–9(e)). In other words, pretreatment with SP can mitigate the loss of NO in the presence of oxidative stress, which might contribute to enhanced EPC survival.

## 4. Discussion

Estrogen deficiency causes oxidative stress and inflammation. Most studies on estrogen deficiency-induced osteoporosis have focused on amelioration of inflammation to promote osteoclast activation in the BM, evaluating the efficacy of therapeutics in terms of bone density [39–42]. Considering that it takes several weeks or months to observe systemic inflammation and bone loss in an animal OVX model, these might deal with bone loss-related cellular/tissue events occurring 2 or 3 months after OVX induction. However, to find a time window to prevent the onset of disease, the initial events after estrogen insufficiency must be analyzed over time.

The early control of age-related immunological/physiological responses allows prevention of the progression of many diseases. The maintenance of vascular function may be the key to reduce the risk of osteoporosis and cardiovascular aging after menopause. Notably, osteoprogenitors are preferentially associated with type H capillaries in the metaphysis owing to the action of various growth factors produced by endothelial cells [43–45]. The angiogenic/paracrine potential and type H vessels were impaired from the early phase after osteoporosis induction. Therefore, the administration of type H vessel inducers to facilitate an increase in type H vessels in the bone defect site may be a promising therapeutic approach to prevent the early onset of osteoporosis [7, 44].

Improvement of type H vessels in estrogen deficiency implies enhancement of the activity/survival of vascular endothelial cells in the absence of estrogen-mediated cellular protection. Estrogen can suppress ROS production by modulating NOX and elevating NO generation through increased expression and activity of eNOS. Thus, oxidative stress can be considered as the principal cause of impaired cellular function and survival under estrogen deficiency. In other words, estrogen loss abrogates cellular antioxidant defense, which might aggravate the loss of type H vasculature by creating excessive ROS. Thus, preservation of type H vessels against oxidative stress is expected to be essential for subsequent osteogenesis [46, 47].

The current study revealed that under OVX conditions, loss of angiogenic potential occurs prior to inflammation and bone density reduction. Insufficient type H vessels and lack of angiogenic growth factors were clearly observed at 4 weeks post-OVX induction, which corresponds to intact bone structure without systemic inflammation. At this time, the collapse of the antioxidants system was observed, showing a reasonable reduction in SOD activity and NO concentration in the BM environment. This confirmed that estrogen deficiency triggers the occurrence of oxidative stress within 4 weeks, and this situation might negatively affect the survival and function of the vascular endothelium in the BM.

Systemic treatment with SP for 4 weeks preserves vascular potential by sustaining angiogenic growth factors, VEGF and PDGF-BB, and type H vessel density, leading to inhibition of bone loss. Thus, SP-mediated vascular protection modulates the extent of bone defects. This emphasizes the role of vascular potential in the maintenance of the bone structure.

SP protects cells against oxidative stress-induced cell death in vitro [29, 30]. In this study, the alteration in SOD activity and NO levels due to OVX induction was clearly reversed by SP treatment. Sufficient bioavailability of NO has beneficial effects on endothelial function and inhibits SOD inactivation [48, 49]. Thus, SP-induced NO enrichment may distinctly contribute to the maintenance of type H vasculature under oxidative stress due to estrogen deficiency. Restoration of SOD activity by SP was observed at 8 weeks post-OVX induction, but its effect was surmised not to be attributed to the direct action of SP (Supplementary Figure 5).

In vitro experiments with BM-EPC showed that the application of SP resulted in sustained cell survival in the presence of ROS. Under these conditions, SP-treated cells had higher NO concentrations than untreated cells. This could be one of the cellular mechanisms through which SP is effective. The critical intervention was that SP treatment was carried out for 4 weeks twice a week after OVX induction, and then it was stopped until 8 weeks. The early modulation of oxidative stress and preservation of vasculature by SP inhibited the onset of bone loss. This means that the early application of SP immediately after estrogen loss conferred its efficacy in OVX.

Additionally, osteoporosis is known to decrease the expression of SP and elevate NK-1R levels in the BM environment, but not in the blood [50, 51]. We previously found that SP concentration was decreased and NK-1R expression was elevated in BM at 8 weeks after osteoporosis induction [17]. In this study, we found a deficiency in SP and increased expression of NK-1R at 4 weeks post-OVX induction (Supplementary Figures 6–8). This environment indicates a lack of SP-NK-1R dynamic signaling in the BM environment under osteoporotic stress. Exogenously injected SP could reach BM and exerts its effect [28, 52]. Thus, it can be inferred that SP administration is likely to compensate for SP deficiency in OVX rats. In conclusion, this study revealed the protective mechanism of SP against osteoporosis in terms of oxidative stress control and vascular preservation. SP modulates oxidative stress to preserve vascular potential, leading to blockage of severe bone loss. SP treatment can be a feasible approach for the treatment of bone defects by targeting type H vessels, and therapeutic application for osteoporosis should be started immediately after menopause, potentially improving the lives of millions of women. Moreover, estrogen loss causes many age-related diseases involving vascular dysfunction. The efficacy of SP on aging-related vascular diseases is an intriguing topic for future study. In order to use SP as a medication, unexpected effects of SP depending on its dosage should be considered and its short half-life is also the crucial point to be checked [28]. The toxicity evaluation of SP in preclinical was completed [53, 54], but administration time of SP should be considered before its administration.

## Data Availability

The datasets used and/or analyzed during the present study are available from the corresponding author on reasonable request.

## Supplementary Materials

Supplementary Materials Supplementary Figure 1: immunostaining for CD31 in BM of OVX rat. OVX was induced in SD rat. 4 and 8 weeks later, the femur was fixed and stained with anti-CD31 antibody to detect Type H vessel. White dot line: growth plate. Supplementary Figure 2: quantification of IL-17 in serum of OVX rat. OVX was induced in SD rat. 4 and 8 weeks later, serum was isolated and then analyzed for IL-17 by ELISA. *p* values of less than 0.05 were considered statistically significant (^∗∗∗^*p* < 0.001). The data are expressed as the mean ± standard deviation (SD) of three independent experiments. Supplementary Figure 3: examination of SOD and GPX in serum of OVX rat. OVX was induced in SD rat. 4 and 8 weeks later, serum was isolated and then analyzed for SOD (A) and GPX (B) activity. *p* values of less than 0.05 were considered statistically significant (^∗∗∗^*p* < 0.001). The data are expressed as the mean ± standard deviation (SD) of the three independent experiments. Supplementary Figure 4: determination of H_2_O_2_ concentration for oxidative stress in EPC. BM-derived EPC was cultured with H_2_O_2_ for 8 h, and then, cell viability was determined by WST-1 assay. *p* values of less than 0.05 were considered statistically significant ^∗∗^*p*. Supplementary Figure 5: examination of SOD and GPX in serum of OVX rat. OVX was induced in SD rat, and SP was injected to the tail vein for 4 weeks. At 8 weeks post OVX induction, serum was isolated and then analyzed for SOD (A) and GPX (B) activity. *p* values of less than 0.05 were considered statistically significant (^∗∗∗^*p* < 0.001). The data are expressed as the mean ± standard deviation (SD) of three independent experiments. Supplementary Figure 6: quantification of SP in serum and BM of OVX rat. OVX was induced in SD rat. At 4 and 8 weeks post OVX induction, serum and BMA were isolated. SP concentration was determined by ELISA. *p* values of less than 0.05 were considered statistically significant (^∗∗∗^*p* < 0.001). The data are expressed as the mean ± standard deviation (SD). BM: bone marrow. Supplementary Figure 7: quantification of SP in BM of OVX rat. OVX was induced in SD rat. At 4 and 8 weeks post OVX induction, the femur was isolated and fixed in formalin. A paraffin-sectioned BM sample was stained for SP. Brown color: reactive area. Supplementary Figure 8: quantification of NK-1R in BM of OVX rat. OVX was induced in SD rat. At 4 and 8 weeks post OVX induction, the femur was isolated and fixed in formalin. A paraffin-sectioned BM sample was stained for NK-1R. Brown color: reactive area.
