# Supplementary material for: Substance P-Mediated Vascular Protection Ameliorates Bone Loss
Source: Oxid Med Cell Longev. 2023 Apr 29;2023:9903336. doi: 10.1155/2023/9903336 (PMC10163975; doi:10.1155/2023/9903336)

Supplementary Figure 1

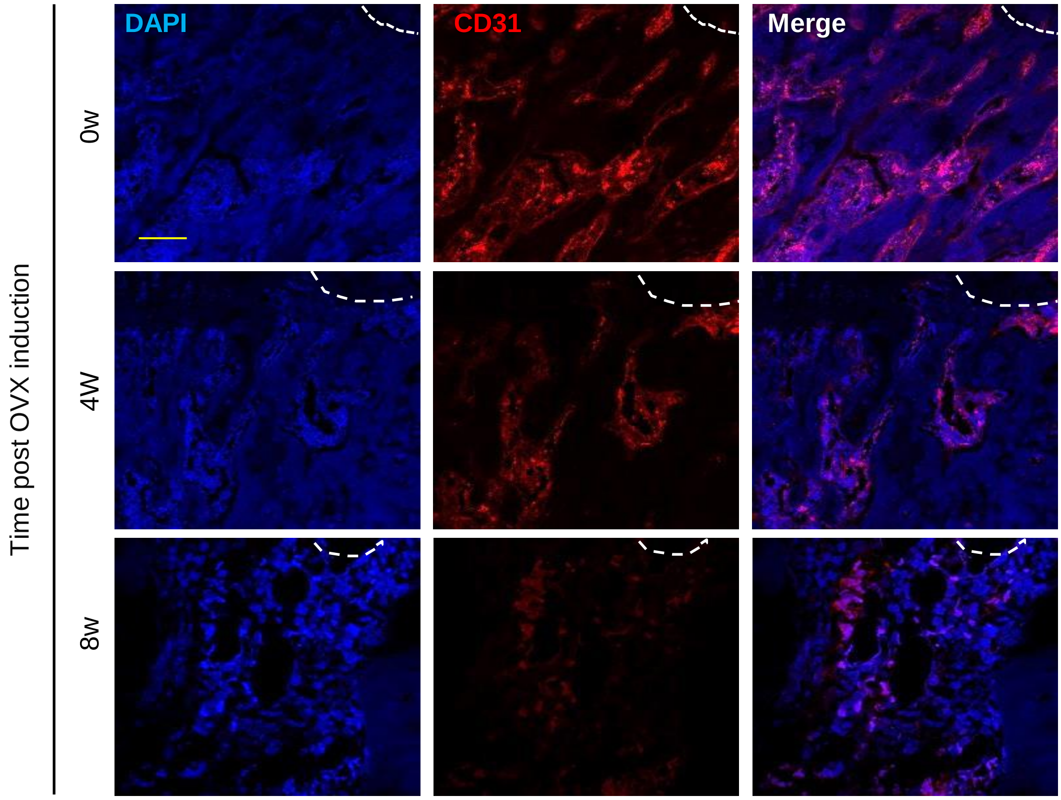

Supplementary Figure 2

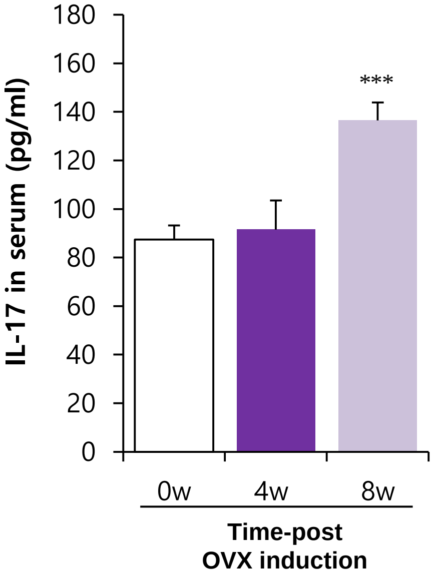

Supplementary Figure 3

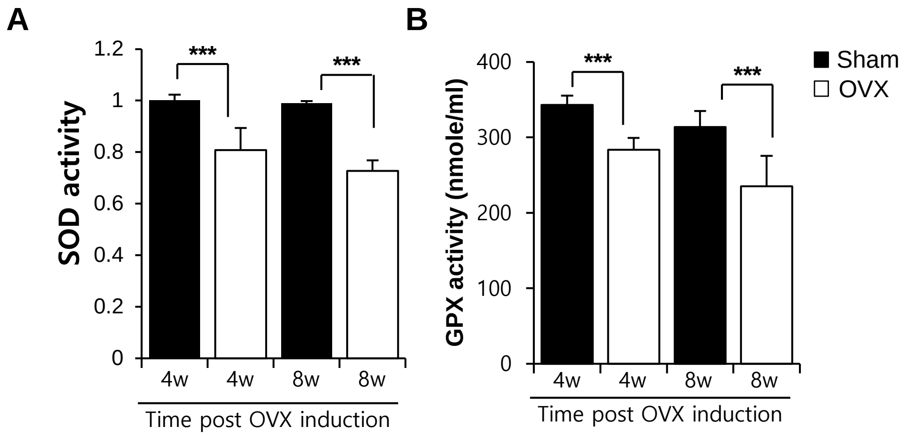

Supplementary Figure 4

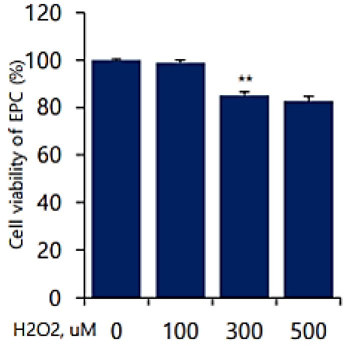

Supplementary Figure 5

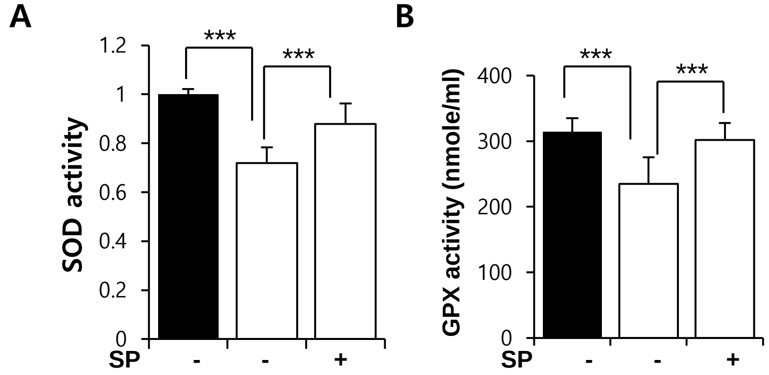

Supplementary Figure 6

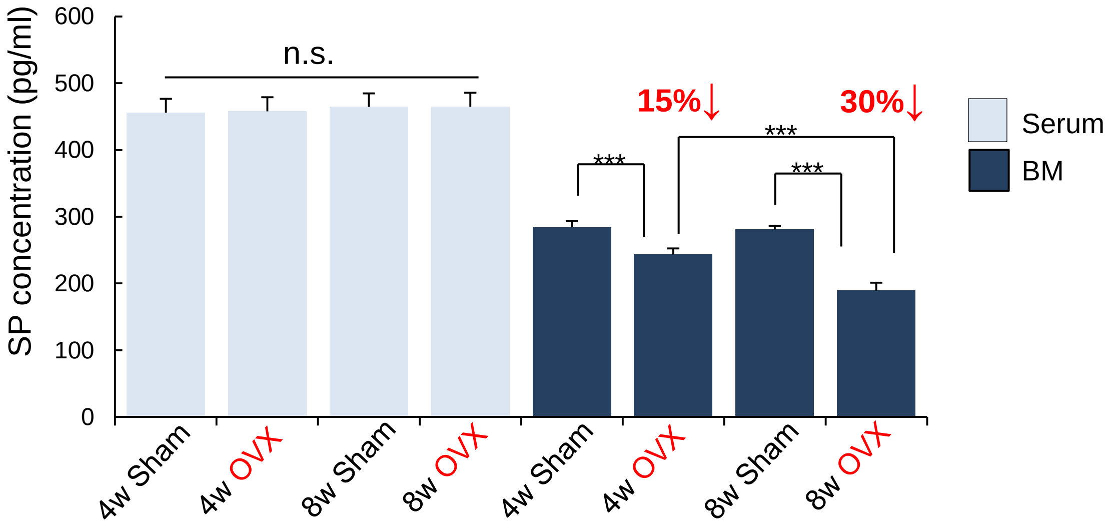

Supplementary Figure 7

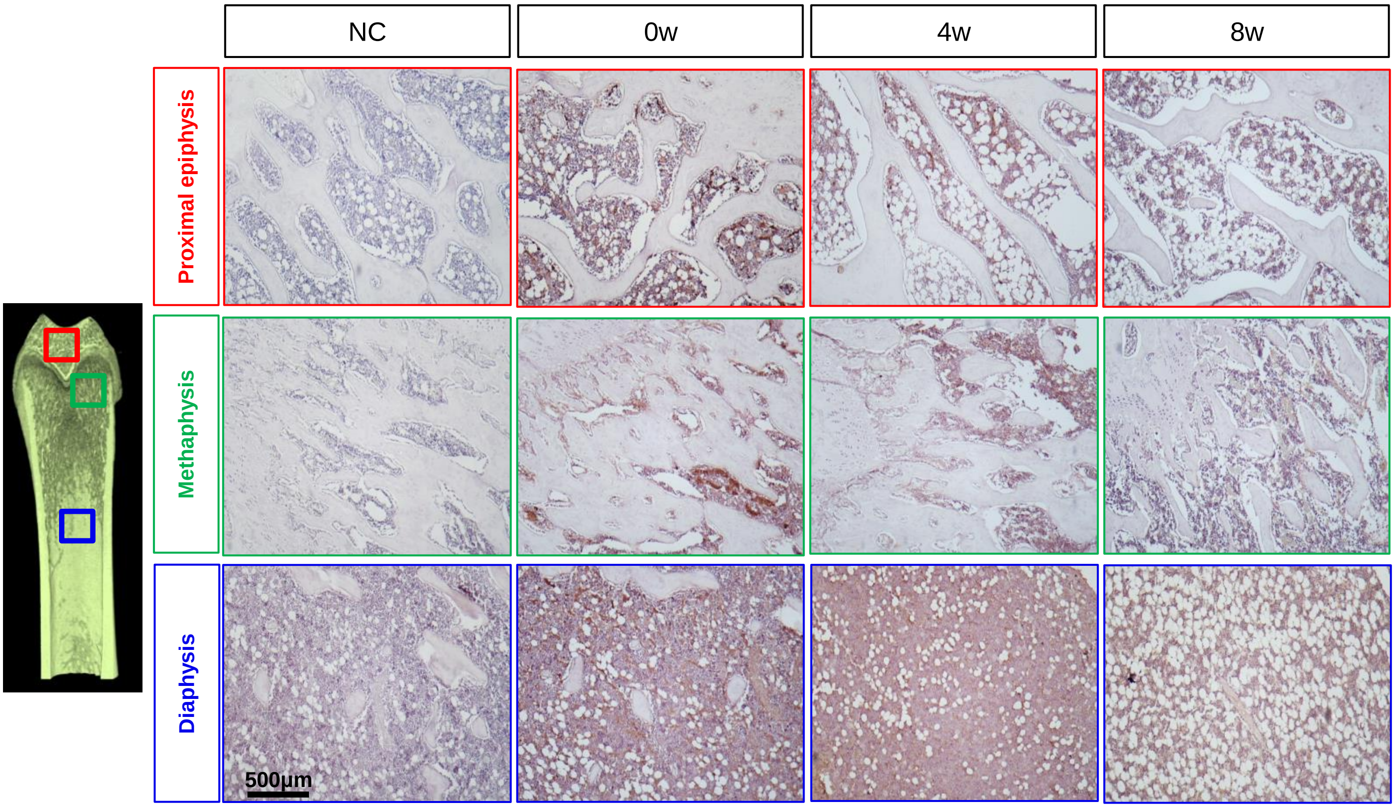

Supplementary Figure 8

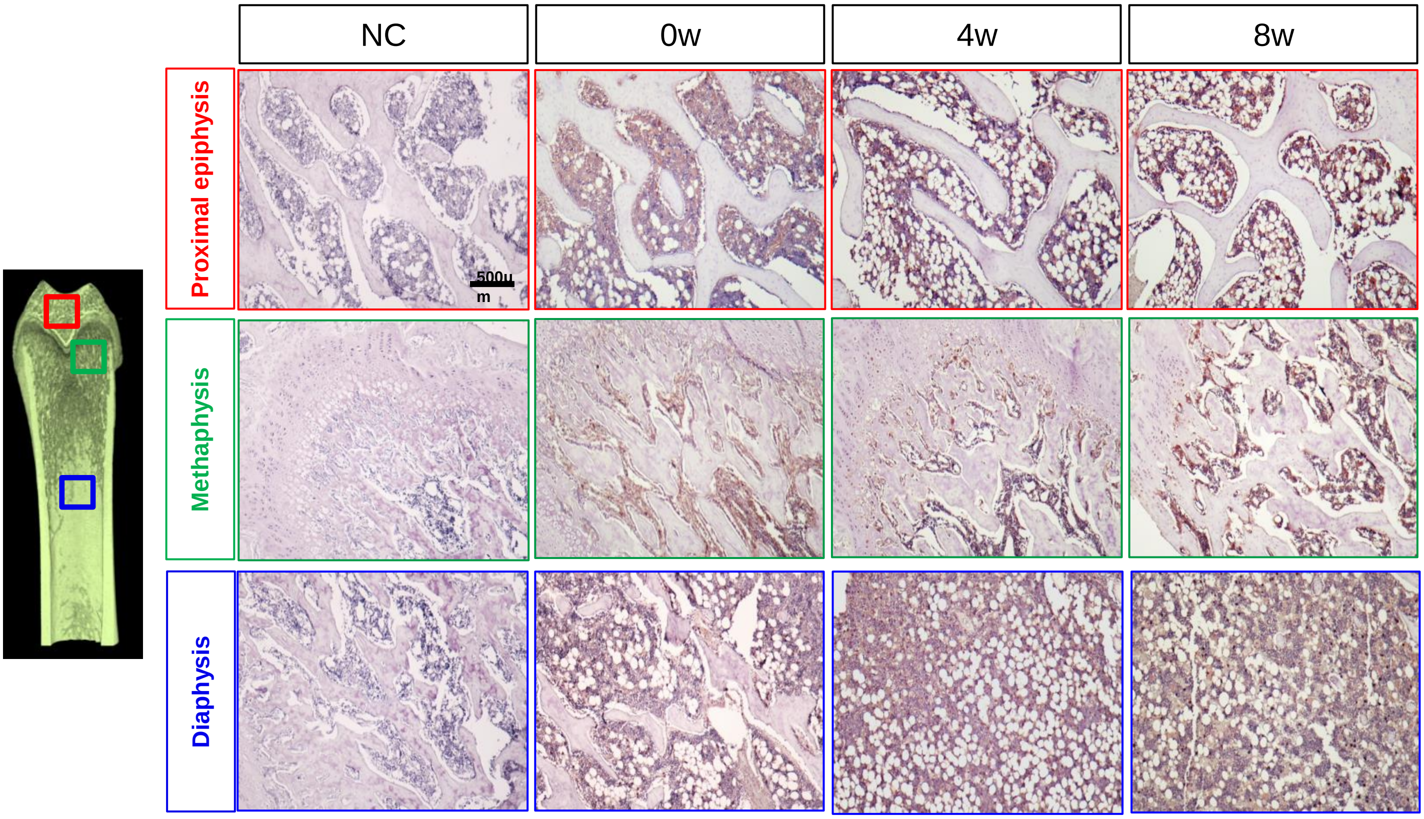

Supplement: Supplementary Materials — Supplementary Figure 1: immunostaining for CD31 in BM of OVX rat. OVX was induced in SD rat. 4 and 8 weeks later, the femur was fixed and stained with anti-CD31 antibody to detect Type H vessel. White dot line: growth plate. Supplementary Figure 2: quantification of IL-17 in serum of OVX rat. OVX was induced in SD rat. 4 and 8 weeks later, serum was isolated and then analyzed for IL-17 by ELISA. p values of less than 0.05 were considered statistically significant (∗∗∗p < 0.001). The data are expressed as the mean ± standard deviation (SD) of three independent experiments. Supplementary Figure 3: examination of SOD and GPX in serum of OVX rat. OVX was induced in SD rat. 4 and 8 weeks later, serum was isolated and then analyzed for SOD (A) and GPX (B) activity. p values of less than 0.05 were considered statistically significant (∗∗∗p < 0.001). The data are expressed as the mean ± standard deviation (SD) of the three independent experiments. Supplementary Figure 4: determination of H2O2 concentration for oxidative stress in EPC. BM-derived EPC was cultured with H2O2 for 8 h, and then, cell viability was determined by WST-1 assay. p values of less than 0.05 were considered statistically significant ∗∗p. Supplementary Figure 5: examination of SOD and GPX in serum of OVX rat. OVX was induced in SD rat, and SP was injected to the tail vein for 4 weeks. At 8 weeks post OVX induction, serum was isolated and then analyzed for SOD (A) and GPX (B) activity. p values of less than 0.05 were considered statistically significant (∗∗∗p < 0.001). The data are expressed as the mean ± standard deviation (SD) of three independent experiments. Supplementary Figure 6: quantification of SP in serum and BM of OVX rat. OVX was induced in SD rat. At 4 and 8 weeks post OVX induction, serum and BMA were isolated. SP concentration was determined by ELISA. p values of less than 0.05 were considered statistically significant (∗∗∗p < 0.001). The data are expressed as the mean ± standard deviation [file 9903336.f1.pdf]
